# Whole Genome Sequencing and Comparative Genome Analysis of the Halotolerant Deep Sea Black Yeast *Hortaea werneckii*

**DOI:** 10.3390/life10100229

**Published:** 2020-10-02

**Authors:** Orazio Romeo, Alessia Marchetta, Domenico Giosa, Letterio Giuffrè, Clara Urzì, Filomena De Leo

**Affiliations:** 1 Department of Chemical, Biological, Pharmaceutical and Environmental Sciences, University of Messina, 98166 Messina, Italy; oromeo@unime.it (O.R.); alemarchetta@unime.it (A.M.); lgiuffre@unime.it (L.G.); urzicl@unime.it (C.U.); 2 Department of Clinical and Experimental Medicine, University Hospital of Messina, 98125 Messina, Italy; dgiosa@unime.it

**Keywords:** *Hortaea werneckii*, black yeasts, whole-genome sequencing, genome assembly, annotation

## Abstract

*Hortaea werneckii*, an extreme halotolerant black yeast in the order of Capnodiales, was recently isolated from different stations and depths in the Mediterranean Sea, where it was shown to be the dominant fungal species. In order to explore the genome characteristics of these Mediterranean isolates, we carried out a de-novo sequencing of the genome of one strain isolated at a depth of 3400 m (MC873) and a re-sequencing of one strain taken from a depth of 2500 m (MC848), whose genome was previously sequenced but was highly fragmented. A comparative phylogenomic analysis with other published *H. werneckii* genomes was also carried out to investigate the evolution of the strains from the deep sea in this environment. A high level of genome completeness was obtained for both genomes, for which genome duplication and an extensive level of heterozygosity (~4.6%) were observed, supporting the recent hypothesis that a genome duplication caused by intraspecific hybridization occurred in most *H. werneckii* strains. Phylogenetic analyses showed environmental and/or geographical specificity, suggesting a possible evolutionary adaptation of marine *H. werneckii* strains to the deep sea environment. We release high-quality genome assemblies from marine *H. werneckii* strains, which provides additional data for further genomics analysis, including niche adaptation, fitness and evolution studies.

## 1. Introduction

*Hortaea werneckii* belongs to the so-called black yeast, a polyphyletic group of pleomorphic and melanised fungi that present in many cases a polyextremotolerant lifestyle [1,2]. Up to date, this species was mainly studied for its remarkable halo-tolerance, being the only known fungus able to grow in a wide range of salinity, from 0 M NaCl to saturation at 5.1 M NaCl [3,4]. For this reason, the fungus is used as a model organism for understanding the osmoadaptation strategies and the molecular mechanisms involved in the tolerance to high salinities of eukaryotic cells [5,6,7,8].

*H. werneckii* is assigned to the division of Ascomycota in the family *Teratosphaeriaceae* (Pezizomycotina, Dothideomycetes, Capnodiales) and the species was previously classified as *Exophiala werneckii*, *Cladosporium werneckii* or *Phaeoannellomyces werneckii* [9], until the genus *Hortaea* was established [10]. The genus *Hortaea* currently also includes the species *Hortaea thailandica*, isolated from *Syzygium siamense* leaf spots in Thailand [11]. The identification at species level is complicated by the fact that some housekeeping genes commonly used for taxonomic purposes, such as β-tubulin, mini-chromosome maintenance protein and translation elongation factor 1, produce ambiguous results in some *H. werneckii* strains [12,13]. So, the sequencing of ribosomal genes together with morphology, biochemical and physiological characteristics is considered the main useful criteria to identify this species [12].

Before 2000, *H. werneckii* was studied mainly for medical interest, as the etiology agent of the human disease “tinea nigra”, a non-invasive skin infection of hands and feet with global distribution but with a higher incidence in tropical and subtropical climates [14,15,16,17]. The physiological preferences of the species, such as an ability to grow better at higher salt conditions at 37 °C, should facilitate skin infection by the fungus [13]. However, the pathogenetic mechanisms are still not clear.

*H. werneckii* is a cosmopolitan fungal species, well adapted to live in hypersaline environments worldwide, such as brine in eutrophic solar salterns, which are considered its primary ecological niche [18]. In fact, it has been shown to be the dominant fungal species in these habitats at salinities above 20% NaCl, and thus its presence was reported in different salty environments such as seawater, beach soils, saltern microbial mats, immersed wood, fish, corals and salt marsh plants [7,18,19,20].

Although *H. werneckii* is not listed as a marine species [21], it is stated that it also occurs in marine habitats, and also in the depths of the oceans [22,23,24].

Recently, *H. werneckii* was also isolated from Mediterranean seawater up to the depth of 3400 m [13,25], where it represented the dominant fungal species. These Mediterranean strains were proven to be highly similar in terms of physiological and genetic characteristics, and significantly deviated from other strains from different sources, being also less halophilic [13]. These findings were explained by the fact that these strains may be less often related to the hypersaline environment of coastal saltpans, suggesting a possible marine origin or evolution in these habitats.

To date, the genomes of 12 *H. werneckii* strains isolated from diverse sources (3 from hypersaline water, 1 from deep-sea water, 1 from rocks, 3 from spider webs, 1 from coast soil and 3 clinical strains) and different countries have been sequenced [26].

The first whole genome sequencing of an *H. werneckii* strain (EXF-2000) isolated from the hypersaline water of salt-pans in Slovenia showed that, differently from related species within the *Dothideomycetes*, the fungus has a diploid genome, characterized by high levels of heterozygosity between the two sub-genomes [27,28]. However, when the genomes of a further 11 *H. werneckii* strains were sequenced, 2 haploid strains (EXF-562 and EXF-2788, isolated from sea coast soil in Namibia and from salterns in Slovenia, respectively) were detected, while the remaining 9 strains, including the strain isolated from the depths of the Mediterranean Sea, were diploid and highly heterozygous, as with the reference genome [26]. Although at least one mating locus was identified in all sequenced strains, the species undergoes clonal reproduction, and the duplication of the genomes seems to be due to intraspecific hybridization events between ancestors [26].

The aim of this study was to sequence the whole genomes of two *H. werneckii* strains isolated from Mediterranean Sea water sampled at 3400 m and 2500 m depths. A de-novo whole genome assembly was carried out for the strain MC873 (3400 m depth), while a re-sequencing of the strain MC848 (2500 m depth) was performed in order to get a better assembly for this strain, as its current draft genome sequence is highly fragmented (5734 contigs; GenBank assembly accession: GCA_003704595.1).

A comparative phylogenomic analysis with other published *H. werneckii* genomes, from different environmental and clinical sources, was also carried out to investigate the evolutionary history and the possible marine origin of the strains from the deep sea.

## 2. Materials and Methods

### 2.1. Fungal Isolates and Genomic DNA Isolation

The genomes of two *H. werneckii* strains, MC848 and MC873, isolated from the stations of Vector and Geostar in the Mediterranean Sea at 2500 and 3400 m depths, respectively [13,25], were examined in this study. Both strains are maintained in the collection of the Department of Chemical, Biological, Pharmaceutical and Environmental Sciences of University of Messina, Italy. Information relative to the *Hortaea werneckii* strains included in this study for comparative genome analysis is reported in Table 1.

For whole-genome sequencing, genomic DNA was extracted from the two marine *H. werneckii* strains after growth on MEA medium for 7 days, using a mechanical glass-beads disruption method followed by traditional phenol\chloroform extraction and ethanol precipitation. Briefly, fungal cells were transferred to a 2 mL eppendorf tube, containing 500 μL of lysis buffer (1% SDS; 0.1 M Tris, pH 8.0; 50 mM EDTA, pH 8.0), 25 μL of NaCl 5 M and about 400 μL of acid washed glass beads (Ø 0.50 mm). The mixture was vortexed for 2 min and centrifuged for 3 min at 14,000 rpm, then the supernatant was collected in a new tube. A volume of 500 μL of lysis buffer was added to the pellet, then vortexed and centrifuged for 3 min at 14,000 rpm. The supernatant was recovered and added to the one previously collected. After addition of 400 μL phenol, the solution was mixed and centrifuged for 5 min at 12,000 rpm and the supernatant was transferred to a new tube with an equal volume of phenol-chloroform (4:1), then the mixture was mixed and centrifuged for 5 min at 12,000 rpm. The supernatant was collected, and the DNA was precipitated with a volume of 1 mL of absolute ethanol and incubated at −80 °C for 1 h, then centrifuged for 10 min at 12,000 rpm. Subsequently the pellet was washed with cold 70% ethanol, dried and dissolved in 50 μL of ultrapure water. RNase treatment was carried out by adding 2 μL of RNase (20 μg/mL) and incubating for 45 min at 37 °C. Finally, the samples were stored at −20 °C. DNA integrity and purity were evaluated spectrophotometrically and by 1.2% agarose gel electrophoresis. High quality DNA (A_260/280_ ≥ 1.8) was used for library construction.

### 2.2. Library Preparation, Sequencing and Genome Assembly

The genomes of the *H. werneckii* MC873 and MC848 strains were sequenced using Illumina NextSeq 500 technology (Illumina, Italy). A total of two sequencing libraries, with insert sizes of approximately 350 bp and 550 bp, respectively, were prepared for each strain and sequenced using the paired-end (2 × 150 bp) strategy. After sequencing, raw reads were preprocessed using the fastpv.0.20.0 software (options: sliding window of 5 bp; average base quality-score of 25; minimum read length of 35 bp) [29] to remove adapters and/or other Illumina technical sequences, including low-quality reads/bases. The final data set, used for genome assembling, contained a total of 70,946,302 (MC873 strain) and 97,637,450 (MC848 strain) quality-controlled reads. Using these clean reads, a de-novo genome assembly was performed using SPAdesv.3.13.0 assembler [30] and the resulting contigs were further processed with SSPACE-Standard v.3.0 software [31] to obtain scaffold sequences. The GapFiller v.1-10 program [32] was then used to close the gaps within pre-assembled scaffolds by reducing the number of undetermined bases in the genome.

### 2.3. Evaluation of the Quality and Completeness of the Genome Assembly and Determination of the Level of Heterozygosity

The quality of genome assemblies was assessed using the QUAST v.5.0.2 program [33] whereas their completeness was quantitatively evaluated with Benchmarking Universal Single-Copy Orthologs (BUSCO) v.3.1.0 [34] by searching for universal single-copy orthologs in different lineage-specific datasets (eukaryota_odb9, fungi_odb9 and ascomycota_odb9) [34]. Finally, the KAT tool v. 2.4.2 [35] and GenomeScope [36] were employed to perform the K-mer frequency (k = 27) analysis and to estimate the level of heterozygosity and duplication of each genome.

### 2.4. Gene Prediction, Functional Genome Annotation and Phylogenomic Analysis

Gene models were predicted separately for each *H. werneckii* genome using the MAKER pipeline (v.3.00.0) [37] combined with the ab-initio gene predictors SNAP (v.2.39) [38] and AUGUSTUS (v. 3.3.3) [39], and further integrated with a full set of reference proteins and expressed sequence tags (ESTs) from Capnodiales (NCBI: txid134362) downloaded from the NCBI Protein and Nucleotide databases, respectively. However, before using these datasets, sequences sharing more than 90% similarity were removed by CD-HIT software [40] in order to reduce the redundancy.

Automatic functional annotation of predicted proteins was performed using PANNZER2 program [41] and resulting proteomes were compared by OrthoFinder (v.2.3.11) (https://github.com/davidemms/OrthoFinder) in order to evaluate the content of orthologous genes and the abundance of distinct orthogroups among the two sequenced strains. An orthogroup was defined as a set of genes derived from a single gene in the last common ancestor of different species. Orthogroups, containing a different number of genes, were subsequently submitted to ShinyGO v0.61 (http://bioinformatics.sdstate.edu/go) to determine if any particular metabolic pathway was enriched in a strain-specific manner.

Finally, the two genome assemblies were also screened for transposable elements (TE) and repetitive DNA sequence content by using Tandem Repeat Finder v.4.09 [42] and RepeatMasker v.4.0.9 (www.repeatmasker.org) softwares. tRNAs were also predicted using the tRNAscan-SE program v.1.3.1 [43].

Phylogenomic analysis was conducted using our two sequenced genomes and additional publicly available *H. werneckii* genome assemblies retrieved from the NCBI BioProject database (accession number: PRJNA428320) and GenBank (assembly accession: GCA_002127715.1) (Table 1). Phylogenetic distance was estimated by the Mash 2.2 toolkit [44] (sketch size, *s*  =  1000; k-mer size, *k*  =  21) while cluster analysis was performed with the pvclustR package [45], using the UPGMA algorithm with a bootstrap analysis of 1000 replicates. The resulting tree was visualized and edited using iTOL (https://itol.embl.de).

## 3. Results

Basic statistics of the *H. werneckii* genome assemblies generated in this study are shown in Table 2. The MC848 and MC873 genomes were sequenced with an average coverage of ~276× and ~207×, respectively, and their predicted size was quite similar, consisting of approximately ~51 Mbp each, with a total GC content of 53.4% (Table 2). The raw reads have been deposited in the Sequence Reads Archive (SRA) database and are available under BioProject identifier (ID) PRJNA641248 (https://www.ncbi.nlm.nih.gov/Traces/study/?acc=PRJNA641248).

**Figure 1:**
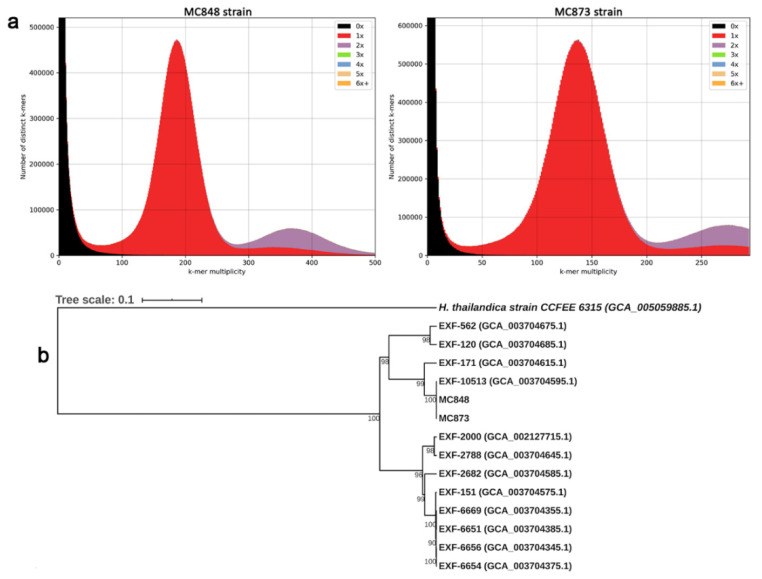
Genome assembly analysis and phylogenomic relationships of *H. werneckii* strains. (**a**) k-mer frequency analysis generated by the KAT tool; plots show KAT spectra of the 27-mer multiplicity of the read set (x axis) against their respective assembly (y axis) for MC873 and MC848 strains. Read content in black represents the 27-mers present in the reads but absent in the assemblies and suggests a fairly complete genome assembly. Red indicates 27-mers are present once in the assembly while purple 27-mers are present twice, indicating that genome duplication events likely occurred; (**b**) Mash-based phylogenetic tree generated using our two *H. werneckii* genome assemblies along with whole-genomes currently available in GenBank database. The code of each strain used is shown to the right of each branch tip and its GenBank accession number. Numbers at the nodes represent percent bootstrap support values based on 1000 replicates.

The draft genome sequences contained a total of 1218 (MC848 strain) and 925 (MC873 strain) assembled contigs (minimum length: ≥200 bp), and have been submitted to the GenBank database under the following accession numbers: JACSRB000000000 (MC848) and JACSRC000000000 (MC873). The comparison of k-mer frequencies to the final assemblies revealed that most of the 27-mers in the reads were represented once in the respective assemblies, consistent with a high-quality genome assembly (estimated assembly completeness: >99.8%) of a predominantly heterozygous microorganism (Figure 1a). This genome-level variability was also confirmed by GenomeScope analysis (k-mer: 27), which showed an extensive level of heterozygosity (average ~4.6%) for both *H. werneckii* genomes.

To examine the phylogenetic relationships among our *H. werneckii* strains and other previously sequenced strains, we generated a Mash-based tree using the whole genomes currently available in GenBank (Figure 1b).

Phylogenomic analysis showed the existence of two main clusters (Figure 1b). The strains from the Mediterranean Sea clustered together, and they were very close to one clinical strain from Brazil (EXF-171) and two environmental isolates—one from salterns of Spain (EXF-120) and one haploid strain from Namibia (EXF-562). The second cluster grouped eight strains: four from the Atacama desert in Chile (EXF-6651, EXF-6654, EXF-6669 and EXF-6656), two clinical strains from Portugal (EXF-151) and from Italy (EXF-2682), and two strains from salterns of Slovenia (respectively, EXF-2000 and the haploid strain EXF-2788).

**Figure 2:**
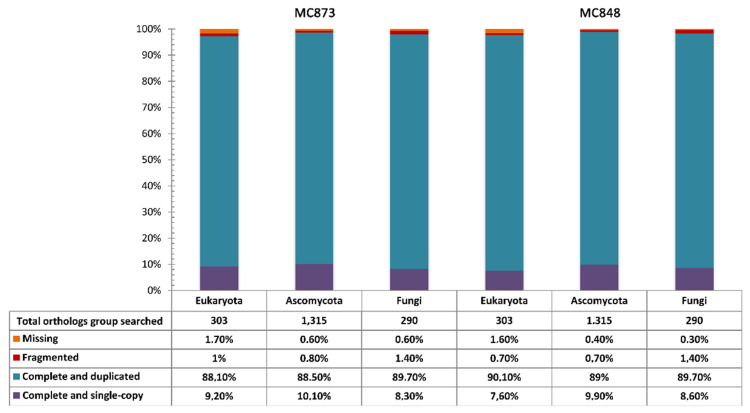
(BUSCO) results showing the number of complete (single and duplicated), missing and fragmented orthologs obtained by searching the Eukaryota, Ascomycota and fungal lineage datasets.

For both MC848 and MC873 genomes, we observed evidence of genome duplication by k-mer frequency analysis (Figure 1a), which was further corroborated byBUSCOanalysis (Figure 2).

Notably, for both *H. werneckii* genomes, we detected a high level of complete and duplicated BUSCO genes (>88%; Figure 2), which is consistent with the level of genome duplication observed. Overall, BUSCO results also confirmed a high level of genome completeness, with over 97% of eukaryotic and fungal genes found to be complete in our assemblies (Figure 2).

Totals of 15,542 and 15,565 genes were predicted in the *H. werneckii* MC873 and MC848 genomes, respectively. There was only a slight difference in protein-coding gene content between the two sequenced genomes (Table 2). Further small variations were also observed for the transposon content (23 extra TE found only in the MC848 strain), especially for the LTR-retrotransposon gipsy and non-LTR LINE-like retrotransposons, including some DNA TE that were exclusively detected in the MC848 strain (Table 3).

Regarding functional analysis of the gene set annotated in this study, we observed small differences among the two *Hortaea* genomes. In fact, over 99% of these genes were assigned to 10,911 unique orthogroups, of which 6052 (55.5%) contained single-copy orthologs, whereas the remaining 4859 (44.5%) included at least 2 genes from a single strain (Supplementary Material Table S1). Interestingly, 1047 orthogroups (1047/10,911;9.6%) showed strain-level differences in the number of genes included in each group. More specifically, of the 1047 orthogroups, 528 (50.4%) were enriched in the MC848 strain and 497 (47.5%) were enriched in the MC873 strain, while the remaining 22 (2.1%) orthogroups included strain-specific genes. In particular, the MC848 strain displayed 10 specific orthogroups containing a total of 20 genes, while the remaining 12 MC873-specific orthogroups included a total of 24 genes (Supplementary Material Table S1). However, most of the strain-specific genes encoded proteins with unknown or still uncharacterized functions (Supplementary Material Table S1).

## 4. Discussion

The recent finding that *H. werneckii* has a ubiquitous distribution in the seawater of the Mediterranean Sea, being the dominant fungal species up to a depth of 3400 m [13,25], has led us to deepen the genomic characterization of these strains to better understand their origin and evolution.

The presence of the fungus was demonstrated also in other deep-sea environments, such as deep-sea hydrothermal ecosystems in the Pacific Ocean [22,24] and sediments at 5000 m depths in the Central Indian Basin [23]. However, the species is not currently considered a marine fungus [21].

Comparative analyses of Mediterranean isolates with other *H. werneckii* strains recovered from different sources evidenced the peculiar genetic and physiological characteristics of the seawater strains compared to the others [13], but these were not sufficient to assign them to another taxonomic group [12].

In this study we carried out a de-novo sequencing of the genome of one strain isolated at a 3,400 m depth (MC873), and a re-sequencing of the genome derived from a 2500 m depth (MC848). The latter strain MC848 was previously sequenced and assembled in 5734 contigs, which were highly fragmented and presumably had an extensive number of errors, especially in terms of total gene number predicted [46].

In this study, a high-quality genome assembly (>99.8%) was obtained for both MC848 and MC873 genomes, which proved to be diploid. In fact, k-mer frequency analysis (Figure 1a) and BUSCO analysis (Figure 2) showed the existence of genome duplication in both genomes. In addition, a high level of heterozygosity (~4.6%) was also detected for both *H. werneckii* genomes. This is a typical hallmark of diploid species and/or eukaryotic lineages that have undergone whole-genome duplication (WGD) by occasional intraspecific hybridization events between two haploid progenitors. This finding was suggested also by Gostinčar et al. [26], who, after analyzing 12 genomes of *H. werneckii*, found that only 2 (EXF-562 and EXF-2788) were haploid, while the remaining strains were diploid and highly heterozygous. Our results support this hypothesis.

Phylogenetic analyses carried out in this study showed the existence of two separate clusters. Interestingly, our strains, recovered from the deep sea at 3400 and 2500 m of depth, but in different stations of the Mediterranean Sea, clustered together, suggesting a degree of environmental specificity for these genotypes (Figure 1b). The placement of our strains in a single well-supported group, together with the previously sequenced EXF-10513 strain (BioSample accession n°: SAMN08295408) (Figure 1b), is consistent with our expectation, as this latter strain and MC848 are the same strain, although strain codes may vary in the publications [26]. However, from the re-sequencing of the genome of this strain (MC848=EXF-10513), we obtained a better assembly with fewer contigs (1218 vs. 5734 contigs), and consequently an improved genome annotation according to other studies [46]. In fact, we detected a lower number of predicted genes (15,410 vs. 17,094) and exons (32,716 vs. 37,837) [26] as expected for less fragmented genome assemblies [46].

Environmental and/or geographical specificity was also observed for other *H. werneckii* genomes in the phylogenetic tree, such as EXF-2000 and EXF-2788 strains, isolated from hypersaline water, or EXF-6651 and EXF-6669 strains, recovered from spider webs in the Atacama desert, Chile [26]. These findings may suggest an evolutionary adaptation of *H. werneckii* strains to such environments.

The sizes of the MC848 and MC873 genomes sequenced in this study were 51,030,830 bp and 50,705,820 bp, respectively, confirming that the DNA content of marine strains may be slightly larger than that of other *H. werneckii* genomes sequenced so far, whose sizes range from 25.2 Mbp to 49.9 Mbp [26].

Genome sizes show dramatic variation among the *Dothideomycetes* [47], and after *Pseudocercospora (Mycosphaerella*) *fijiensis* (genome size ~74.14 Mbp) [47], *H. werneckii* possesses the largest genome among members of the order of Capnodiales [28] (www.zbi.ee/fungal-genomesize). However, additional large genomes are frequently described in other dothideomycetous black fungi, such as *Friedmanniomyces endolithicus* (genome size 46.75 Mbp), isolated from the extreme environment of the Antarctic [48].

Large scale genome duplication in fungi seems to be associated with selective advantage in terms of stressful environmental conditions. In *Rhizopus oryzae* and *Phycomyces blakeesleeanus* species, WGD contributes to pathogenicity and the expansion of signal transduction and light sensing [49,50], while increases in genome size were also observed in experimental evolution studies in *Saccharomyces cerevisiae*, when the yeast is exposed to stressful concentrations of salt and UV radiations [51,52]. In fact, genome duplication provides the raw material for further evolution processes. In the case of *H. werneckii*, the large genome also contains expanded gene families encoding metal cation transporters [27] that are supposed to confer a selective advantage in hypersaline environments [28].

The results obtained in this study support the recent hypothesis that most *H. werneckii* strains are likely derivatives from intraspecific hybridization and, due the phylogenomic differences observed between strains from different sources, we could suppose that marine strains are evolving in this environment where they are well adapted.

The release of high-quality genome assemblies from marine *H. werneckii* strains provides additional data enabling further genomics analysis, including niche adaptation, fitness and evolution studies for investigating the diversification of *Hortaea* species and their specific associations with stressful and harsh environments.

## Figures and Tables

**Table 1:** Strains of *Hortaea werneckii* included in this study.

Strain	Other Collection	Source	Country	Reference
EXF-2000	CBS 100457	Hypersaline water of salt-pans	Slovenia	[28]
EXF-2788		Hypersaline water of salt-pans	Slovenia	[26]
EXF-120		Hypersaline water of salt-pans	Spain	[26]
EXF-562		Soil on the sea cost	Namibia	[26]
EXF-171	CBS 111.31	Human, *Keratomycosis*	Brazil	[26]
EXF-2682	CBS 126.35	Human, *Trichomycosis nigra*	Italy	[26]
EXF-151	CBS 107.67	Human, *Tinea nigra*	Portugal	[26]
EXF-6651		Spider web, Atacama desert	Chile	[26]
EXF-6654		Spider web, Atacama desert	Chile	[26]
EXF-6669		Spider web, Atacama desert	Chile	[26]
EXF-6656		Rock in a cave, Atacama desert	Chile	[26]
EXF-10513	MC848 V2500 b	Seawater of Mediterranean Sea; 2500 m depth, Vector station	Italy	[26]; This study
MC873	Geo f 100 c	Seawater, Mediterranean Sea; 3400 m depth, Geostar station	Italy	This study

**Table 2:** Overall assembly statistics and gene content of the two *H. werneckii* genomes sequenced in this study.

*H. werneckii* Genome Features	MC873 Strain	MC848 Strain
Total number of sequenced bases	10,481,489,013	14,036,248,558
Total number of reads with Q-score ≥25	70,784,397	98,305,008
Total number of mapped reads (%)	70,711,190 (99.9%)	95,135,691 (96.8%)
Unmapped reads	73,207 (0.1%)	3,169,317 (3.2%)
Number of total contigs	925	1218
Number of contigs >1 Kbp	761	612
Largest contig (bp)	604,832	576,474
Genome size (bp)	50,705,820	51,030,830
Average coverage depth	~207×	~276×
GC content (%)	53.4	53.4
Total number of predicted genes	15,542	15,565
Protein-coding genes	15,397	15,410
tRNAs	126	132
snRNAs	5	5
Total gene length (bp)	26,021,394	26,184,209
Longest gene (bp)	15,052	19,877
Number of exons	32,681	32,716
Longest exon (bp)	12,966	12,966
Exon average length (bp)	~742	~746
Gene average length (bp)	~1690	~1699
Tandem Repeat Number	13,188	13,315
Simple repeats	11,183	11,282
Low-complexity repeats	2005	2033

**Table 3:** Classes of transposable elements (TE) and their relative numbers detected in the two sequenced marine *H. werneckii* genomes.

TE Class	MC848	MC873	TE Class	MC848	MC873
DNA (notclassified)	2	3	LINE I-Jockey	1	1
DNA CMC-EnSpm	2	3	LINE L1	4	3
DNA Dada	10	8	LINE L1-Tx1	4	3
DNA Ginger-1	2	2	LINE L2	1	0
DNA hAT	1	2	LINE RTE	1	0
DNA hAT-Ac	18	17	LINE RTE-BovB	3	5
DNA hAT-Charlie	2	2	LTR (notclassified)	3	3
DNA Kolobok-T2	1	0	LTR Copia	8	1
DNA Merlin	1	0	LTR ERV1	7	6
DNA-Maverick	0	1	LTR ERVK	6	8
DNA Mule-MuDR	1	1	LTR Gypsy	165	151
DNA P	1	1	LTR Ngaro	12	12
DNA TcMar-ISRm11	1	1	LTR Pao	18	17
DNA-TcMar-Tc1	0	1	RC Helitron	1	2
LINE CR1	1	0	SINE B2	1	1
LINE I	1	0	SINE-tRNA-RTE	0	1
Total				279	256

## Data Availability

The Illumina raw reads have also been submitted into the Sequence Read Archive (SRA) database and are associated with the BioProject ID: PRJNA641248. The draft whole-genome sequences of strains MC848 and MC873 have also been deposited at DDBJ/ENA/GenBank under accession numbers JACSRB000000000 andJACSRC000000000, respectively. The versions described in this article are the first versions JACSRB000000000 and JACSRC000000000.

## Supplementary Materials

The following are available online at https://www.mdpi.com/2075-1729/10/10/229/s1, Table S1: Overall statistics of all orthogroups detected in the two *Hortaea werneckii* strains.
